# The Spectra of Pathogenic Variants and Phenotypes in a Chinese Cohort of 298 Families with Osteogenesis Imperfecta

**DOI:** 10.3390/genes16040416

**Published:** 2025-03-31

**Authors:** Siji Zhou, Xiuzhi Ren, Yixuan Cao, Huan Mi, Mingchen Han, Lulu Li, Chendan Jiang, Yuqian Ye, Chaoqun Zheng, Binshan Zhao, Tao Yang, Nan Wu, Zhen Li, Lingqian Wu, Xiuli Zhao

**Affiliations:** 1 State Key Laboratory for Complex, Severe, and Rare Diseases, Department of Medical Genetics, Institute of Basic Medical Sciences, Chinese Academy of Medical Sciences, School of Basic Medicine, Peking Union Medical College, 5# Dongdan San Tiao, Beijing 100005, China; sijizhou@ibms.pumc.edu.cn (S.Z.);; 2 Key Laboratory in Science and Technology Development Project of Suzhou (CN), Pediatric Orthopedics, Children’s Hospital of Soochow University, No. 92 Zhongnan Street, Suzhou Industrial Park, Suzhou 215025, China; 3 Department of Orthopedic Surgery, Peking Union Medical College Hospital, Peking Union Medical College and Chinese Academy of Medical Sciences, 1 Shuai Fu Community, Dongcheng District, Beijing 100730, China; 4 Department of Stomatology, Peking Union Medical College Hospital, Peking Union Medical College and Chinese Academy of Medical Sciences, 1 Shuai Fu Community, Dongcheng District, Beijing 100730, China; 5 Center for Medical Genetics, Hunan Key Laboratory of Medical Genetics & Hunan Key Laboratory of Animal Models for Human Diseases, School of Life Sciences, Central South University, No. 172 Tongzipo Road, Changsha 410017, China; 6 Center for Rare Diseases, State Key Laboratory of Complex, Severe, and Rare Diseases, Peking Union Medical College Hospital, Chinese Academy of Medical Sciences, 1 Shuai Fu Community, Dongcheng District, Beijing 100730, China

**Keywords:** osteogenesis imperfecta, genotype–phenotype correlation, Chinese cohort, variant spectrum, clinical phenotypes

## Abstract

**Background:** Osteogenesis imperfecta (OI) is marked by clinical and genetic heterogeneity, and the genotype–phenotype correlation remains not very clear. We conducted a clinical and genetic study in a Chinese OI cohort to determine the spectra of phenotypes and pathogenic variants. **Methods:** In this study, 298 Chinese families were recruited from 2019 to 2024. Clinical phenotypes including fractures, short stature, skeletal deformities, blue sclera, dentinogenesis imperfecta, and hearing loss were recorded and analyzed. Next-generation sequencing combined with PCR-based techniques was used to detect candidate pathogenic variants. Variant pathogenicity was evaluated via conservation analysis, bioinformatics analysis, and functional studies at the cellular level. In this OI cohort, the spectra of pathogenic variants, clinical phenotypes, and genotype–phenotype correlations were analyzed. **Results:** Our OI cohort included 71 type I (23.83%), 122 type III (40.94%), 90 type IV (30.20%), and 15 type V (5.03%) probands. The cohort consisted of 196 children (65.77%) and 102 adults (34.23%). For the first time, phenotypic differences between different age groups were confirmed. In total, we identified 231 variants, including 47 novel pathogenic variants. Notable variants include two atypical splicing variants, one small deletion, two small duplications, one gross deletion, and one gross duplication. New genotype–phenotype correlations were observed: patients with *SERPINF1* variants had the highest fracture frequency, followed by those with *WNT1* variants, compared to patients with other gene variants. **Conclusions:** We performed the clinical and genetic analysis in a large Chinese OI cohort. The expanded spectra of genetic variants and clinical phenotypes were constructed by identifying 47 novel pathogenic variants and summarizing the skeletal and extra-skeletal manifestations. The current paper will provide important evidence for the precise diagnosis of the disease.

## 1. Introduction

Osteogenesis imperfecta (OI) is a monogenic connective tissue disorder characterized by reduced bone mass, frequent fractures, short stature, and limb deformities [1,2,3]. This condition affects approximately 1/15,000 to 1/20,000 newborns [4,5]. Some OI patients also exhibit extra-skeletal manifestations, including blue sclerae, dentinogenesis imperfecta (DI), hearing loss, ligamentous laxity, cardiac valve abnormalities, and pulmonary function impairment [6,7].

Previous studies have shown that 80%–85% of OI cases are caused by abnormal synthesis and processing of type I collagen due to heterozygous variants in the genes encoding the α1 (*COL1A1*) or α2 (*COL1A2*) chains of type I collagen, which are inherited in an autosomal dominant (AD) pattern [5,8]. Fewer than 5% of OI cases result from a hotspot variant (c.−14C>T) in *IFITM5*, while the remaining autosomal recessive (AR) and X-linked patients account for approximately 10% of all OI patients [8]. At least 20 non-type I collagen genes—*IFITM5*, *SERPINF1*, *CRTAP*, *P3H1*, *PPIB*, *SERPINH1*, *FKBP10*, *PLOD2*, *BMP1*, *SP7*, *TMEM38B*, *WNT1*, *CREB3L1*, *TENT5A*, *MESD*, *LRP5*, *KDELR2*, *CCDC134*, *SPARC*, and *MBTPS2*—have been implicated in the development of OI [9,10,11,12,13,14,15,16,17,18]. Variants in *COL1A1*/*2* lead to OI by reducing the synthesis or altering the structure of type I collagen [19]. In contrast, variants in non-type I collagen genes affect processes such as procollagen post-translational modification, helical folding, osteoblast differentiation, and bone matrix mineralization [20].

Although over 20 genes responsible for OI have been reported, the pathogenic genes of some patients remain unclear due to rare variant types, and the correlation between genotype and phenotype is not well understood. Hitherto, numerous clinical genetic studies on OI populations have been conducted globally, revealing distinct clinical and genetic characteristics of OI across different ethnic groups [21,22,23,24,25]. Since 2012, our laboratory has been dedicated to clinical genetics studies on the Chinese OI population, establishing the spectra of pathogenic gene variations and clinical phenotypes [26,27,28]. Previous studies on several Chinese populations have revealed significant differences in the major OI pathogenic genes, the spectra of genetic variations and clinical phenotypes, and the prevalence of hot variations between Chinese and other ethnic groups [29]. However, the genetic and clinical diversity of Chinese OI populations remains underexplored, particularly in terms of genotype–phenotype correlations.

In this study, we enrolled 298 OI families to update and expand the clinical and genetic spectra of the Chinese OI population. We are committed to establishing an expanded spectrum of pathogenic variants, improving genotype–phenotype correlations, and discovering the unique genetic characteristics of the Chinese OI population.

## 2. Methods

### 2.1. Editorial Policies and Ethical Considerations

This study was approved by the Institutional Review Board of the Institute of Basic Medical Sciences, Chinese Academy of Medical Sciences, Beijing, China (Approval No. 015-2015), on 11 March 2015. Informed consent was obtained from all adult participants, as well as from the legal guardians of children.

### 2.2. Participants and Clinical Analysis

In this study, 298 Chinese families suspected of having OI were recruited from 2019 to 2024. The inclusion criteria for patients included experiencing at least two fractures per year, or obvious limb deformities, or vertebral compression fractures, and all patients with low bone density. Individuals with metabolic disorders, such as hyperthyroidism or hypophosphatemic rickets disease, were excluded from the cohort by serum biochemical testing. Clinical data, medical history, and blood or tissue samples were collected from the probands and their family members. The ages of the patients at their first fractures and last visits were recorded. Height was converted to a Z-score specific to age and sex, based on reference data for Chinese children aged 0–18 years [30].

To compare disease severity between pediatric and adult patients, fracture frequency was evaluated using the average number of fractures per year from the first to the last fractures. For statistical analysis, sclerae with atypical colors such as gray or blue gray were considered blue. The laxity of ligaments and joint mobility were determined by examination of joint dorsiflexion. DI was diagnosed by dentists based on the established clinical and radiographic criteria. Spinal deformities such as kyphosis, scoliosis, and lordosis were diagnosed and recorded by X-ray examination. Additionally, patients who required a wheelchair, crutches, or assistance from others to move were recorded as being unable to walk independently.

The OI cohort was divided into five clinical types: types I–IV followed the traditional Sillence classification based on severity and clinical characteristics [31]. Specifically, type I was the mildest form, characterized by blue sclerae and minimal fractures; type II was more severe, often leading to early death; type III was the most severe among postnatal patients; and type IV fell between type I and type III in terms of severity. Type V was characterized by calcification of the interosseous membrane and growth of a hyperplastic callus at fracture sites [32,33]. Type I patients were further classified into subtypes IA and IB based on the presence or absence of DI. All families were assigned distinct ID numbers, such as “PUMC-OI-001”.

### 2.3. Nucleic Acid Isolation

Genomic DNA was isolated using a conventional proteinase K-phenol-chloroform method from peripheral blood, villi, amniotic fluid, or the umbilical cord blood of fetuses. Total RNA was extracted using TRIzol reagent (Invitrogen, Carlsbad, CA, USA). DNA and RNA were evaluated using a NanoPhotometer N60 (Implen, Munich, Germany) and used for genetic analysis.

### 2.4. Next-Generation Sequencing (NGS)

Genomic DNA (1–3μg) was randomly fragmented, end-repaired, phosphorylated, and ligated with paired-end adaptors. The size distribution was assessed and sequenced on a HiSeq 4000 System (Illumina, Inc., San Diego, CA, USA). The sequencing image files were subjected to base calling and filtering to generate high-quality clean data. Next-generation sequencing, including whole-exome sequencing (WES) or whole-genome sequencing (WGS), was conducted on each proband. The raw data from NGS were aligned to the human genome reference sequence (hg19). Tools were used for file sorting and duplicate marking. Variant calling was used to identify single-nucleotide polymorphisms and indels, and variant information was annotated using various databases. Synonymous single-nucleotide variants or those with > 1% minor allele frequency in the 1000 Genomes database were discarded based on the filtering strategy.

HGMD (https://www.hgmd.cf.ac.uk/ac/; Professional April 2023)), ClinVar (http://www.clinvar.com/; accessed on 15 May 2019), the International Thousand Talents Genome Project database (http://www.internationalgenome.org/; accessed on 22 September 2019), dbSNP (https://ngdc.cncb.ac.cn/databasecommons/; accessed on 29 February 2020), and gnomAD (http://www.gnomad-sg.org/; accessed on 10 July 2020) were used to determine whether any identified variant was novel. If the variant had been reported, its pathogenicity was relatively clear. The pathogenicity of novel variants was predicted by bioinformatics analysis and validated by functional experiments at the cellular level if necessary.

### 2.5. Polymerase Chain Reaction and Sanger Sequencing

The candidate pathogenic variants found in NGS, and familial co-segregation of the genotype and phenotype, were validated by polymerase chain reaction (PCR) combined with Sanger DNA sequencing. Sanger sequencing was performed using the ABI 3730XL DNA Analyzer (Thermo Fisher Scientific, Waltham, MA, USA). Genomic DNA, cDNA, and the amino acid reference sequences of *COL1A1* (NM_000088.3 and NP_000079.2), *COL1A2* (NM_000089.3 and NP_000080.2), *IFITM5* (NM_001025295.2 and NP_001020466.1), *SERPINF1* (NM_002615.5 and NP_002606.3), *CRTAP* (NM_006371.4 and NP_006362.1), *SERPINH1* (NM_001235.3 and NP_001226.2), *FKBP10* (NM_021939.3 and NP_068758.3), *WNT1* (NM_005430.3 and NP_005421.1), and *PLOD2* (NM_182943.2 and NP_891988.1) were obtained from the University of California, Santa Cruz (UCSC) Genome Browser database (http://genome.ucsc.edu/; accessed on 3 November 2020) and the National Center for Biotechnology Information (NCBI) Reference Sequence Project. Primers were designed using the online tool Primer3 (http://primer3.ut.ee/; accessed on 18 January 2020) and inspected using the UCSC Genome Browser BLAT and In-Silico PCR online tools. All primer sequences can be found in our previous publication [21]. Genomic DNA was amplified using LA Taq polymerase with GC Buffer (TaKaRa Bio, Dalian, China), and the PCR volumes were set based on the manufacturer’s instructions [21]. The annealing temperature was 58 °C for all PCR reactions. The candidate pathogenic variants were verified in the probands and their available family members.

### 2.6. Reverse Transcription-PCR and Minigene Assay

The candidate splicing variants located in exons or deep introns were verified by reverse transcription-PCR (RT-PCR) or minigene assay. RNA was extracted from the in vitro cultured skin fibroblasts from patients. The PrimeScript™ RT Reagent Kit with gDNA Eraser kit (TaKaRa Bio, Cat. # RR047A) was used for reverse transcription following the manufacturer’s instructions. Alterations in the RNA sequences were identified by RT-PCR and Sanger DNA sequencing. If the patient’s tissue was not available, a minigene assay was performed to identify the aberrant splicing. The procedure for minigene assay included vector construction, transfection, in vitro expression, RNA extraction, RT-PCR, and DNA sequencing, as described previously [27]. The target fragments comprised at least the intron/exon in which the variant was located and its flanking introns and exons. The expression vectors were constructed by ligating the target fragment into a linearized pCAS2 vector using *Hind*III and *EcoR*I restriction endonucleases (New England Biolabs, Ipswich, MA, USA). *Escherichia coli* DH5α competent cells (TaKaRa Bio, Dalian, China) were used for transformation. HEK293T cells were transiently transfected with purified recombinant vectors via the Invitrogen Lipofectamine 3000 Transfection Kit (Thermo Fisher Scientific, Waltham, MA, USA). RNA was extracted from HEK293T cells 24 h after transfection, followed by RT-PCR and Sanger sequencing.

### 2.7. Identification of Gross Deletions and Duplications

Multiplex ligation-dependent probe amplification (MLPA) and quantitative PCR (qPCR) were used to detect gross deletions and duplications. For typical OI patients, in whom *COL1A1* and *COL1A2* variants had been excluded by DNA sequencing, MLPA was used to detect gross deletions/duplications in *COL1A1* and *COL1A2* following the manufacturer’s instructions using probemixes P271 (B2–0412) and P272 (B2–0412) (MRC Holland, Amsterdam, The Netherlands), respectively [26]. The PCR amplicons from MLPA were separated by capillary electrophoresis using the Applied Biosystems 3730 × l DNA Analyzer (Thermo Fisher Scientific, Waltham, MA, USA), and the data were analyzed using Coffalyser (version 140721.1958; MRC Holland). For patients with only one mutant allele identified in AR genes, qPCR was performed to validate the presence of a potential large deletion or duplication in the other allele. Breakpoints involving genome structure variants were confirmed by the combination of WGS, Gap-PCR, and DNA sequencing. Primer sequences were listed in Table S2.

### 2.8. Statistical Analysis

Only probands were used in the statistical analyses. The results were presented as means ± standard deviations (SDs) for normally distributed data (e.g., height Z-score or age) and as percentages for binomially distributed data (e.g., the status of DI or blue sclerae). Differences between two or more groups were evaluated by independent-sample Student’s *t*-test or one-way analysis of variance (ANOVA), as appropriate. Welch’s *t*-test or the Brown–Forsythe test was considered appropriate when Levene’s test indicated heteroscedasticity in the Student’s *t*-test or ANOVA, respectively. Pearson’s chi-square test or Fisher’s exact test was used to analyze contingency tables, if appropriate. A *p* value less than 0.05 was considered statistically significant. All statistical analyses were conducted using SPSS Statistics (version 25.0; IBM, New York, NY, USA).

## 3. Results

### 3.1. Clinical Characteristics

**Figure 1:**
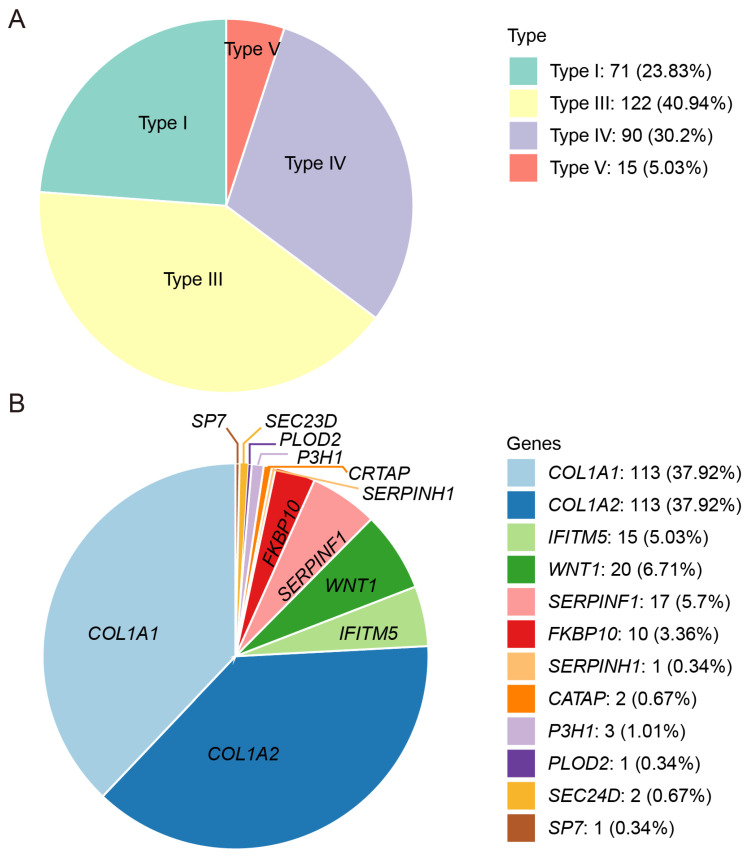
Patient composition of the OI cohort in this study. (**A**). Distribution of OI patients with different clinical subtypes. (**B**). Distribution of OI patients with different pathogenic genes.

This study enrolled 298 Chinese families with OI, comprising 113 familial cases (37.92%) and 185 sporadic cases (62.08%). The cohort included 196 child probands (65.77%) and 102 adult probands (34.23%). The probands were grouped according to the current OI clinical classification: 71 of type I, 122 of type III, 90 of type IV, and 15 of type V (Figure 1A). However, no type II OI patients were included in our cohort. Patients manifested typical OI features, including frequent fractures, short stature, and limb deformities. The mean number of fractures was three, with 13.83% of the probands (39 of 282) having over thirty fractures and 3.55% (10 of 282) suffering from more than a hundred fractures. The median height Z-score was −4.45 (n = 262), and 67.56% (177 of 262) had short stature, defined as a height Z-score < −2.00. Other clinical features included scoliosis (24.64%, 71 of 288), an inability to walk independently (51.94%, 107 of 206), blue sclerae (82.14%, 230 of 280), DI (66.18%, 182 of 275), and hearing loss (16.12%, 39 of 242).

Clinical characteristics were observed among the different OI types. Type I patients were the least susceptible to scoliosis (5.97%) and the inability to walk independently (9.76%) and were more likely to have blue sclerae (93.94%) compared to other OI types. Additionally, type I patients were divided into subtypes IA (n = 36) and IB (n = 30) based on the presence or absence of DI. The onset of fractures occurred later in type IA patients than in type IB patients (7.80 ± 11.72 vs. 2.58 ± 3.96; *p* = 0.016). Type III patients exhibited a greater number of fractures (30.94 ± 31.21, *p* = 0.042). Additionally, type III patients were more prone to scoliosis (40.34%), an inability to walk independently (75.79%), and DI (81.03%) compared to the other types. The severity of type IV was similar to that of type V. However, type V patients were the least likely to develop DI (40%) (Table 1).

Significant differences in phenotypes were observed between child and adult OI patients. Adult patients exhibited more severe phenotypes in stature (Z-score, −6.23 ± 4.81 vs. −3.24 ± 3, *p* < 0.001) and the number of fractures (26.65 ± 28.98 vs. 13.28 ± 19.03, *p* < 0.001) compared to children. Additionally, the adults exhibited a higher prevalence of extra-skeletal phenotypes, including scoliosis (34% vs. 19.68%, *p* = 0.007), DI (74.75% vs. 61.36%, *p* = 0.024), hearing loss (30% vs. 7.90%, *p* < 0.001), and blue sclerae (89.58% vs. 78.26%, *p* = 0.019), compared to children (Table S3).

### 3.2. Genetic Characteristics

Genomic analysis of the 298 OI families achieved a 100% detection rate, identifying 231 pathogenic variants in total. These 231 variants identified in this OI cohort were distributed among 12 OI-related genes and comprised eight variant types: missense, nonsense, splicing, frameshift, in-frame, regulatory, gross deletion, and gross duplication (Figure 1B). Of these variants, ninety-two (39.83%) were found in *COL1A1*, ninety-two (39.83%) in *COL1A2*, fifteen (6.49%) in *SERPINF1*, twelve (5.19%) in *WNT1*, ten (4.33%) in *FKBP10*, and two (0.87%) in each of *SEC24D*, *P3H1*, and *CRTAP*. Only one variant was detected in each of the following: *IFITM5*, *SP7*, *SERPINH1,* and *PLOD2*. Apart from *COL1A1* and *COL1A2*, the genes with the highest number of detected variants, in descending order, were *SERPINF1*, *WNT1*, and *FKBP10* (Table S1).

Of the 231 pathogenic variants, 47 were novel variants, distributed across *COL1A1* (n = 23), *COL1A2* (n = 17), *WNT1* (n = 2), *SERPINF1* (n = 1), *FKBP10* (n = 2), *CRTAP* (n = 1), and *SP7* (n = 1) (Table 2). The novel variants comprised twenty missense, ten frameshift, two in-frame, and thirteen intronic variants leading to alternative splicing.

### 3.3. Notable Variants

**Figure 2:**
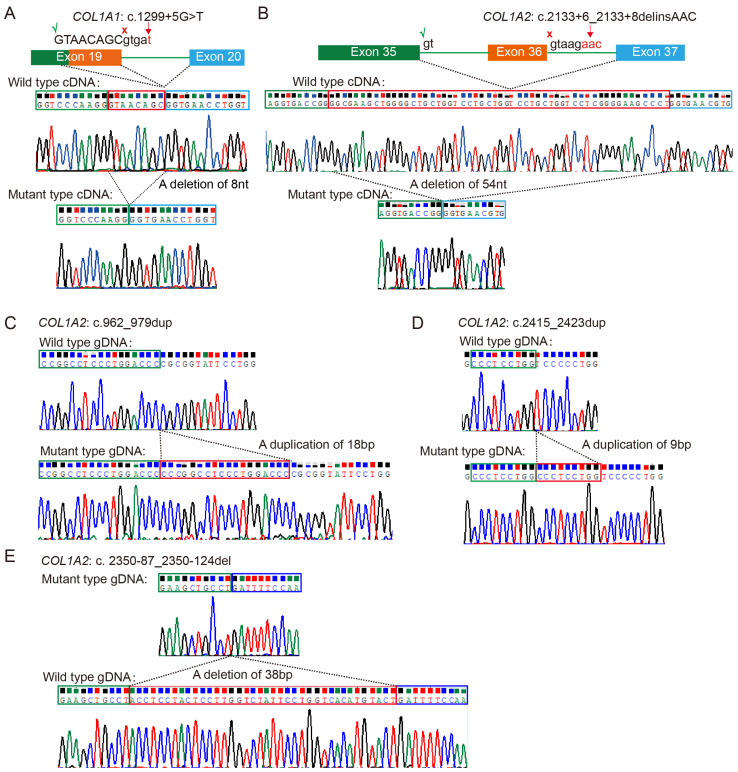
Identification of five rare variants in the OI cohort. (**A**,**B**) Atypical splicing caused by deep intronic variants *COL1A1*: c.1299+5G>T and *COL1A2*: c.2133+6_2133+8delinsAAC. Red crosses (×) indicate the original splicing sites and green checkmarks (√) indicate the new splicing sites induced by the pathogenic variants. (**C**,**D**) The duplications c.962_979dupCCCGGCCTCCCTGGACCC(p.Arg327Profs*1047) and c.2415_2423dupCCCTCCTGG(p.Pro810_Pro812dup) in *COL1A2* were confirmed by cloning sequencing. (**E**) A deletion variant of c.2350-87_2350-124del in *COL1A2* was validated by cloning sequencing. All the red boxes in this figure indicate the deleted/duplicated sequences in mRNA or DNA.

An atypical splicing variant in Family PUMC-OI-41 (*COL1A1*: c.1299+5G>T) was identified, which had been missed in the previous WES test. Minigene assays and Sanger sequencing demonstrated that the *COL1A1*: c.1299+5G>T variant caused a splice site alteration, leading to a truncated exon 19 with an 8-nucleotide deletion (GTAACAGC) in the mature transcript (Figure 2A). Additionally, another splicing variant was identified in Family PUMC-OI-145 (*COL1A2*: c.2133+6_2133+8delinsAAC). This variant caused exon 36 skipping in the *COL1A2* transcript, confirming its pathogenic effect (Figure 2B).

In addition, two duplications [*COL1A2*: c.962_979dupCCCGGCCTCCCTGGACCC (p. Arg327Profs*1047) and *COL1A2*: c.2415_2423dupCCCTCCTGG (p. Pro810_Pro812dup)] and one deletion (*COL1A2*: c.2350-87_2350-124del) were identified in Families PUMC-OI-137, PUMC-OI-199, and PUMC-OI-147, respectively. These mutant alleles were validated by T-cloning sequencing (Figure 2C–E).

**Figure 3:**
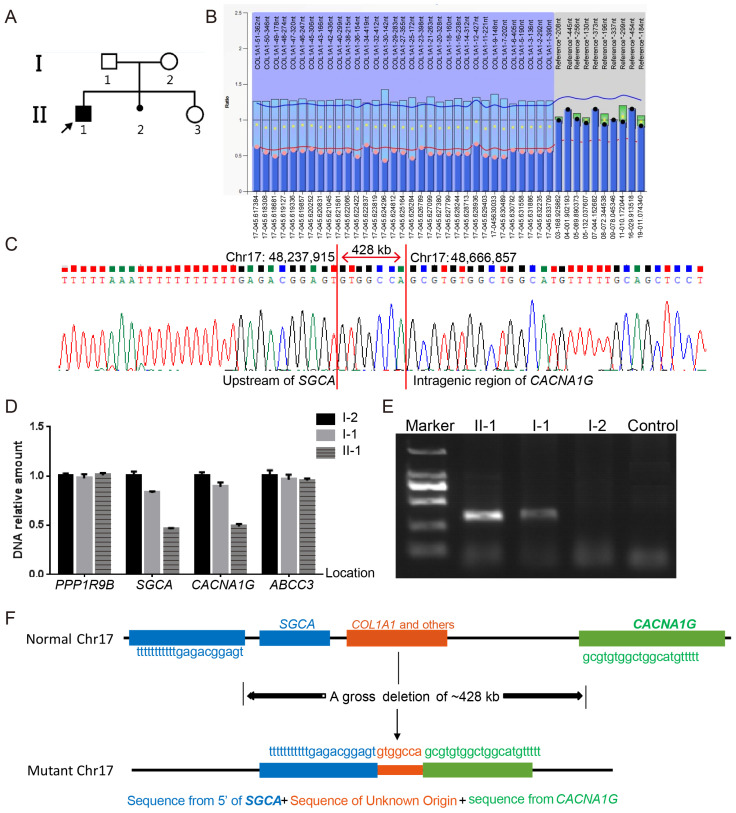
Identification of a gross deletion in Family PUMC-OI-109. (**A**) Pedigree of the family. Note: arrow, proband; solid square, OI patient. (**B**) A heterozygous deletion of the entire *COL1A1* gene was identified in the proband (II-1) by MLPA. The normalized probe ratio thresholds were defined as follows: diploid (black circles; 0.7 ≤ ratio ≤ 1.3) and deletions (pink circles; ratio < 0.7). (**C**) Breakpoint analysis demonstrated a fusion between the upstream region of *SGCA* and the intragenic region of *CACNA1G*, which was mediated by a non-homologous sequence. (**D**) qPCR analysis confirmed the deletion boundaries involving *SGCA* and *CACNA1G*. The genes in the gross deletion are arranged in the following sequence from 5′ to 3: *PPP1R9B*-*SGCA*-*CACNA1G*-*ABCC3*. (**E**) Gap-PCR results indicated the large deletion in the proband (II-1) and father(I-1) but not in the mother (I-2) or normal control. The DNA marker was DL2000. (**F**) Schematic diagram of the gross deletion (chr17:48,237,915-48,666,858, hg19), spanning from the upstream of *SGCA* to the intragenic region of *CACNA1G*.

**Figure 4:**
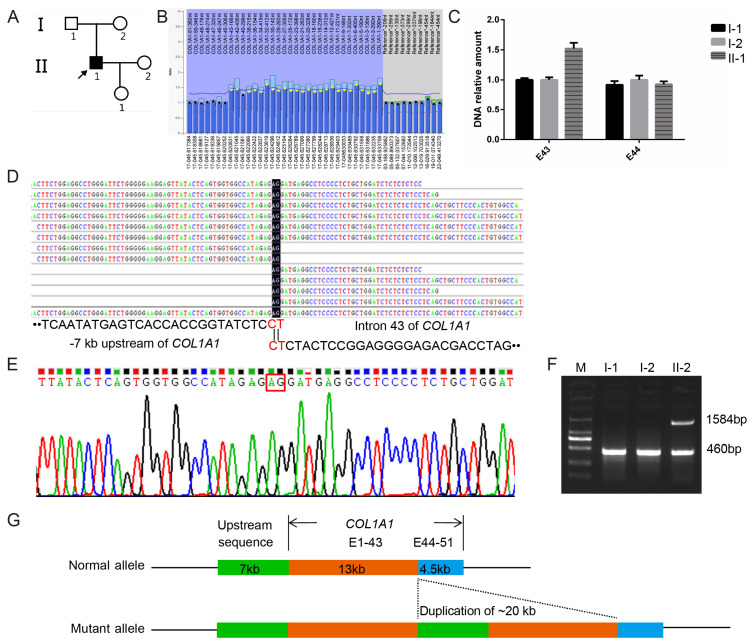
Identification of a novel gross duplication in Family PUMC-OI-110. (**A**) Pedigree structure of the family. Note: arrow, proband; solid square, OI patient. (**B**) A duplication spanning exons 1–43 of *COL1A1* was detected in the proband by MLPA analysis; The normalized probe ratio thresholds were defined as follows: diploid (black circles; 0.7 ≤ ratio ≤ 1.3) and duplication (yellow circles; ratio >1.3). (**C**) The duplication identified in the proband via MLPA was validated in the core family (I-1, I-2, and II1) through quantitative analysis of exons 43 and 44 in *COL1A1*. (**D**) WGS read coverage of the breakpoint junction aligned with the 7 kb upstream region (left) and exon 43 (right) of *COL1A1*. (**E**) The breakpoint junction was validated by DNA Sanger sequencing. (**F**) Gap-PCR confirmed that the gross duplication was a de novel variant. (**G**) Schematic diagram of the gross duplication (chr17:48,286,224-48,265,650, hg19), and the green area represents a 7kb region upstream of *COL1A1*, the tangerine orange area encompasses exons 1 to 43 of *COL1A1*, and the blue area covers exons 44 to 51 of *COL1A1*.

Probands PUMC-OI-109 and PUMC-OI-110 exhibited typical OI phenotypes (Figure 3A and Figure 4A). Pathogenic variants were ruled out from all OI candidate genes by WES and Sanger sequencing. MLPA analysis revealed that PUMC-OI-109 was heterozygous for a deletion of the entire *COL1A1* gene, while PUMC-OI-110 was heterozygous for a duplication spanning exons 1 to 43 (Figure 3B and Figure 4B). To determine the breakpoints of the gross deletion and duplication, WGS, Gap-PCR, and Sanger sequencing were performed on both probands. Proband PUMC-OI-109 exhibited a deletion of 428.943 kb situated within the region chr17:48237915-48666858 (hg19), encompassing 13 genes (*SGCA*, *HILS1*, *COL1A1*, *TMEM92*, *XYLT2*, *MRPL27*, *EME1*, *LRRC59*, *ACSF2*, *CHAD*, *RSAD1*, *MYCBPAP*, *EPN3*, *SPATA20*, and *CACNA1G*). Proband PUMC-OI-109 was heterozygous for the deletion while his father was mosaic for the same deletion detected by qPCR and Gap-PCR (Figure 3D,E). These findings suggested that the deletion had been inherited from the father. Precise sequencing of the breakpoint revealed a 20 kb duplication (chr17:48286224-48265650, hg19) in Proband PUMC-OI-110, who was heterozygous for this duplication. The duplication was absent in his parents (Figure 4C–G). These findings suggested that the duplication in PUMC-OI-110 was either a de novo variant or due to germline mosaicism in one of the parents.

### 3.4. Genotype–Phenotype Correlations

Genotype-specific phenotypic correlations were identified in this OI cohort. Patients with *SERPINF1* variants had the highest fracture frequency (7.04 ± 6.99 per year, *p* < 0.001), followed by those with *WNT1* variants (3.39 ± 3.56 per year), compared to patients with other gene variants. Additionally, a comparison of 241 AD OI patients with 57 AR OI patients revealed that AR patients tended to exhibit a more severe fracture than AD patients. In contrast, AR patients were less likely to exhibit blue sclerae (43.64% vs 91.56%, *p* < 0.001) and hearing loss (2.44% vs. 18.91%, *p* = 0.009) compared to AD patients (Table 3).

In patients with aberrant collagen type I, correlations were observed between variant types and phenotypes. Compared to the patients with a quantitative alteration (n = 32), those with a structural defect (n = 162) exhibited shorter statures (Z-score, −5.52 ± 4.32 vs. −1.13 ± 2.01; *p* < 0.001), more fractures (19.44 ± 25.32 vs 11.82 ± 12.52, *p* = 0.014), a higher prevalence of DI (76% vs. 50%, *p* = 0.004), and a reduced ability to walk independently (56.76% vs. 21.05%, *p* = 0.004). Compared to patients with type I procollagen defect caused by non-glycine substitutions, those with glycine substitutions exhibit more severe short statures (Z-score, −5.38 ± 4.36 vs. −2.49 ± 2.75; *p* < 0.001), scoliosis (27.1% vs. 12.9%, *p* = 0.025), and a later onset of fractures (6.79 ± 8.44 vs. 4.67 ± 5.68 years, *p* = 0.034) (Table 3). Moreover, phenotypic differences were observed between adult and child patients. Among patients with *COL1A1/COL1A2* variants, the affected children exhibited milder short statures and fewer total fractures, but a higher fracture frequency compared to the adults (Table S3).

## 4. Discussion

In this study, we recruited 298 Chinese OI families and identified 47 novel pathogenic variants. We expanded the spectra of genetic variants and phenotypes, revealing novel genotype–phenotype correlations and identifying associations between the phenotype and patient age at the first visit.

The probands in this study included 71 type I (23.83%), 122 type III (40.94%), 90 type IV (30.20%), and 15 type V (5.03%) patients. Compared to the studies of other teams, our cohort showed a lower proportion of type I patients (23.83%) than those reported in Dutch (58%), Russian (54.7%), UK (41.03%), Japanese (64.15%), and Italian (71.98%) cohorts [23,34,35,36,37,38]. In this cohort, we identified 231 variants in 12 causative genes, involving eight variant types, thereby expanding the OI variant spectrum (Table S1). Compared to studies on OI populations conducted by other groups, the most prevalent pathogenic genes in this Chinese OI cohort were also *COL1A1* and *COL1A2*. However, the most frequently observed recessive OI pathogenic genes in our cohort were *WNT1*, *SERPINF1*, and *FKBP10*, differing from those of other research groups. The patients with abnormal type I collagen constituted 75.84% (226/298) of the cohort, a lower proportion than those reported in Sweden (97.01%), Vietnam (90%), and Brazil (88.4%) [25,39,40,41]. Because some patients in this study had previously been screened for the common variants in *COL1A1*/*COL1A2* at other hospitals, our OI cohort included a high proportion of patients with non-type I collagen variants and rare pathogenic variants.

In this study, the proportion of patients with *COL1A1* and *COL1A2* variants was comparable, each accounting for 37.92% (113/298). *COL1A1* variants were predominantly missense and splicing variants, particularly glycine substitutions and typical splicing variants, which are typically associated with more severe clinical phenotypes [34]. Nonsense variants and gross deletions/duplications in *COL1A1* were linked to milder clinical manifestations mediated by haploinsufficiency mechanisms, although these variants were less prevalent [42]. In contrast, variants in *COL1A2* consisted mainly of missense variants, the majority of which were glycine substitutions. The variant spectrum of *COL1A1* encompasses nearly all types of variants, whereas nonsense variants, classical splicing variations, and gross deletions/duplications are quite rare in the variant spectrum of *COL1A2*. These observations have not been systematically reported in previous studies [34,35,41]. We believe that patients with *COL1A2* variants resulting in functional deficits may only exhibit a certain degree of osteoporosis, rather than presenting as OI patients due to attenuated skeletal phenotypes [43].

Compared to the populations of other studies, our cohort included more individuals with variants in non-type I collagen genes such as *IFITM5*, *SERPINF1*, *WNT1*, and *FKBP10* [23,34,35,36,37,38]. The *IFITM5*: c.-14C>T variant was the sole pathogenic variant identified in all OI type V families [44]. Homozygous or compound heterozygous *WNT1* variants were the most common genotypes in this Chinese AR OI cohort (n = 20, 6.71%), followed by *SERPINF1* (n = 17, 5.71%) and *FKBP10* (n = 10, 3.36%), consistent with our previous findings [28].

Abnormalities in type I collagen include both qualitative and structural variants [45]. We identified more probands with structural variants than qualitative variants (162 vs. 32), and structural variants were associated with more severe phenotypes, such as short stature, more fractures, inability to walk, developmental delays, and DI (Table 3). Qualitative variants (haploinsufficiency) are associated with a milder OI phenotype, whereas mild OI resulting from structural abnormalities of type I collagen is seldom seen [46]. This pattern aligns with the findings of our research. Conversely, structural variants in type I collagen, such as glycine substitutions, were associated with more severe symptoms, primarily observed in type III OI patients [9]. Notably, the frequency of DI varies significantly across different studies, yet consistently correlates with clinical types. Ventura, et al. found that the detection rates of DI in OI type I, type III, and type IV were 9.8–31%, 56–86%, and 36–71%, respectively [47], which were similar to the statistical results in our cohort (Table 1).

Most deep intronic splice variants are associated with milder phenotypes [27]. Probands PUMC-OI-41 (*COL1A1*: c.1299+5G>T) and PUMC-OI-145 (*COL1A2*: c.2133+6_2133+8delinsAAC) both exhibited the phenotypes like OI type I, characterized by normal statures, blue sclerae, and recurrent femur fractures (≤10 times) (Table S1). These findings were consistent with our previous results [27]. Additionally, Probands PUMC-OI-137 (*COL1A2*: c.962_979dupCCCGGCCTCCCTGGACCC) and PUMC-OI-199 (*COL1A2*: c.2415_2423dupCCCTCCTGG) also exhibited mild symptoms (type I OI). The former is a nonsense variant caused by a frameshift variant resulting from duplication, while the latter is an in-frame duplication. In contrast, PUMC-OI-147, with a deletion of the intron in *COL1A2* (c.2350-87_2350-124del), exhibited severe clinical symptoms (type III OI), including extremely short statures (Z-score, −12.87) and high-frequency fractures (≥ 60 times) (Table S1). Furthermore, two probands (PUMC-OI-109 and PUMC-OI-110) with gross deletion or duplication in *COL1A1* displayed mild phenotypes due to haploinsufficiency. Overall, most patients with haploinsufficiency mechanisms, including deep intronic variants, splicing variants, and gross deletions/duplications, exhibited milder clinical manifestations (OI type I or type IV).

AR OI patients were more likely to exhibit severe skeletal phenotypes than AD patients, although a small proportion of AR patients presented extra-skeletal manifestations such as blue sclerae, DI, and hearing loss [48]. Patients with *SERPINF1* variants exhibited the highest fracture frequency and were more prone to severe deformities but were less likely to present blue sclerae and DI (Table 3). Moreover, disease severity in *WNT1* patients was milder than that in *SERPINF1* patients. *FKBP10* patients exhibited the shortest statures (−5.97 ± 4.41), the most severe deformity (40%), and the highest prevalence of DI (71.43%) but were less likely to have blue sclerae (11.11%). Phenotypes are related to both the type of variation and the pathogenic gene involved [29].

Significant differences in phenotype were observed between child and adult OI patients, overall and within various clinical types and different genotypes (Table 1 and Table S3). Among patients with *COL1A1/COL1A2* variants, the children exhibited milder short statures, an earlier age at first fracture, and fewer total fractures but a higher fracture frequency compared to the adults. Notably, these children also had a lower prevalence of hearing loss. In addition, children with *IFITM5* variants (type V OI) exhibited a higher fracture frequency compared to the adult group (2.75 ± 2.32 per year vs. 0.87 ± 0.45 per year, *p* = 0.027) (Table S3). However, age is currently not utilized as a reference indicator for the diagnosis, treatment, and early warning of OI. After a multicenter validation, the stature data obtained from this study will provide a clinical reference for predicting the height of children with OI [49,50,51]. Therefore, the data pertaining to age, phenotype, and genotype will provide novel insights into enhancing the diagnosis, classification, prognosis, and treatment of OI.

## 5. Conclusions

This comprehensive clinical and genetic analysis of a large Chinese OI cohort revealed an expanded spectrum of 231 pathogenic variants, including 47 novel variants. By integrating genetic and clinical data, we identified new genotype–phenotype correlations and significant associations between age and phenotype. The variant spectrum of OI established in our cohort not only provides evidence for genetic counseling and prenatal diagnosis for OI patients but also broadens the horizons for further investigation into the pathogenic mechanisms and gene therapy of OI. In summary, this study holds significant importance for advancing high-level clinical practice and scientific research in OI in the future.

## Figures and Tables

**Table 1:** Clinical phenotype characteristics of the OI cohort.

Individuals (n)		Age (Year) Mean ± SD	Height Z-Score Mean ± SD	Age at First Fracture (Year) Mean ± SD	Number of Fractures (n) Mean ± SD	Frequency of Fractures (n/Year) Mean ± SD	Scoliosis (Yes) %	Walking Ability (Yes)%	Blue Sclerae (Yes) %	DI (Yes) %	Hearing Loss (Yes) %
Total	298	15.385 ± 11.703	−4.454 ± 4.085	5.54 ± 7.494	17.88 ± 23.756	2.603 ± 3.286	24.643	48.059	82.143	66.182	16.116
Female	151	15.9 ± 11.441	−5.067 ± 4.39	5.468 ± 7.658	18.928 ± 25.447	2.212 ± 2.541	27.586	48.039	83.453	67.153	18.033
Male	147	14.859 ± 11.982	−3.802 ± 3.638	5.58 ± 7.337	16.833 ± 21.977	2.99 ± 3.858	21.678	47.573	80.851	65.217	14.167
*p* value		0.444	**0.012**	0.899	0.46	**0.046**	0.245	0.947	0.57	0.734	0.413
Children	195	8.053 ± 4.302	−3.237 ± 3.003	2.457 ± 2.896	13.284 ± 19.027	3.101 ± 3.811	19.681	54.412	78.261	61.364	7.895
Adult	102	29.402 ± 7.978	−6.629 ± 4.814	11.345 ± 9.759	26.647 ± 28.976	1.646 ± 1.528	34	34.783	89.583	74.747	30
*p* value		-	**<0.001**	**<0.001**	**<0.001**	**<0.001**	**0.007**	**0.008**	**0.019**	**0.024**	**<0.001**
Type I	71	11.754 ± 12.137	−2.115 ± 2.8	5.455 ± 9.337	4.682 ± 2.763	1.888 ± 2.387	5.97	90.244	93.939	54.545	10.909
Type III	122	19.721 ± 10.907	−6.828 ± 4.456	5.793 ± 6.679	30.935 ± 31.206	2.886 ± 3.52	40.336	24.211	83.051	81.034	21.905
Type IV	90	12.778 ± 10.554	−2.958 ± 2.125	5.123 ± 6.385	10.783 ± 8.19	2.794 ± 3.634	16.092	54.386	72.84	58.974	11.765
Type V	15	12.7 ± 12.349	−2.627 ± 3.252	5.997 ± 10.664	11.214 ± 9.2	2.348 ± 2.19	33.333	58.333	73.333	40	14.286
*p* value		0.426	0.155	0.938	**0.042**	0.121	**<0.001**	**<0.001**	**0.008**	**<0.001**	0.196
Type I											
I A	36	14.224 ± 13.185	−2.736 ± 3.297	7.802 ± 11.717	5.111 ± 3.059	1.754 ± 2.127	2.857	100	97.143	0	13.793
I B	30	9.556 ± 11.087	−1.369 ± 1.881	2.577 ± 3.96	4.214 ± 2.378	2.011 ± 2.752	10	80	89.655	100	7.692
*p* value		0.123	0.056	**0.016**	0.192	0.686	0.232	**0.035**	0.218	**<0.001**	0.469

Significant differences (*p* < 0.05) in bold font. DI, dentinogenesis imperfecta; SD, standard deviation.

**Table 2:** Forty-seven novel pathogenic variations identified in the OI cohort.

Gene	Nucleic Acid Change	Distribution of Variants	Amino Acid Change	Effects
*COL1A1*	c.122dupT	exon 2	Glu42Thrfs*8	Frameshift
	c.182G>A	exon 2	Cys61Tyr	Missense
	c.261dupC	exon 2	Glu88Argfs*81	Frameshift
	c.370-1G>C	intron 4	-	Splicing
	c.545delG	exon 7	Gly182Valfs*83	Frameshift
	c.868G>C	exon 13	Gly290Arg	Splicing
	c.904-2A>T	intron 13	-	Splicing
	c.1201G>C	exon 19	Gly401Arg	Missense
	c.1299+5G>T	intron 19	-	Splicing
	c.1886G>C	exon 28	Gly629Ala	Missense
	c.2354delG	exon 34	Gly785Valfs*323	Frameshift
	c.2714G>C	exon 39	Gly905Ala	Missense
	c.2795delT	exon 39	Gly926Valfs*182	Frameshift
	c.2929G>A	exon 40	Gly977Ser	Missense
	c.2938-1G>C	intron 40	-	Splicing
	c.3068C>T	exon 42	Ser1023Phe	Missense
	c.3008delC	exon 42	Pro1003Leufs*105	Frameshift
	c.3433G>A	exon 47	Gly1145Ser	Missense
	c.3679dupC	exon 48	Arg1227Profs*2	Frameshift
	c.4309_4357dup	exon 51	Glu1453Alafs*114	Frameshift
	c.4325_4326del	exon 51	Val1442Glyfs*108	Frameshift
	chr17: 48237915_48666858	whole gene	-	Gross deletion
	chr17: 48286224_48265650	exon 1-43	-	Gross duplication
*COL1A2*	c.739-1g>c	intron 15	-	Splicing
	c.792G>T	exon 16	Lys264Asn	Splicing
	c.812G>T	exon 17	Gly271Val	Missense
	c.866G>A	exon 17	Gly289Asp	Missense
	c.962_979dupCCCGGCCTCCCTGGACCC	exon 19	Arg327Profs*1047	Frameshift
	c.2133+8a>c	intron 35	-	Splicing
	c.2350-87_2350-124del	intron 38	-	Splicing
	c.1207G>A	exon 22	Gly403Ser	Missense
	c.1297_1300del5	exon 23	433_435del	In-frame
	c.1324G>C	exon 23	Gly442Arg	Missense
	c.1657G>C	exon 28	Gly553Arg	Missense
	c.2179G>C	exon 36	Gly727Arg	Missense
	c.2359G>A	exon 39	Gly787Ser	Missense
	c.2405G>C	exon 40	Gly802Ala	Missense
	c.2415_2423dupCCCTCCTGG	exon 40	Pro810_Pro812dup	In-frame
	c.2783G>T	exon 43	Gly928Val	Missense
	c.2846G>C	exon 44	Gly949Ala	Missense
*WNT1*	c.403G>T	exon 3	Val135Phe	Missense
	c.918-3C>G	intron 4	-	Splicing
*SERPINF1*	c.151G>A	exon 2	Val51Met	Splicing
*FKBP10*	c.812_813del2	exon 5	Glu271Aspfs*101	Missense
	g.41818629_41824066del5438	from intron 4	-	Splicing
*CRTAP*	c.471+3delA	intron 1	-	Splicing
*SP7*	c.38G>T	exon 2	Gly13Val	Missense

**Table 3:** The genotype–phenotype correlations of the OI cohort.

Individuals (n)		Age (Year) Mean ± SD	Height Z-Score Mean ± SD	Age at First Fracture (Year) Mean ± SD	Number of Fractures (n) Mean ± SD	Frequency of Fractures (n/Year) Mean ± SD	Scoliosis (Yes) %	Walking Ability (Yes) %	Blue Sclerae (Yes) %	DI (yes) %	Hearing Loss (Yes) %
*COL1A1*	113	16.875 ± 12.161	−4.119 ± 4.516	5.818 ± 7.131	15.84 ± 20.324	2.354 ± 3.312	21.905	55.556	96.078	63.366	24.444
*COL1A2*	113	17.425 ± 11.61	−5.054 ± 3.81	6.53 ± 8.404	19.16 ± 24.892	2.052 ± 1.787	24.107	45.679	89.815	77.778	14.433
*IFITM5*	15	12.7 ± 12.349	−2.627 ± 3.252	5.997 ± 10.664	11.214 ± 9.2	2.348 ± 2.19	33.333	58.333	73.333	40	14.286
*WNT1*	20	6.708 ± 6.617	−5.065 ± 3.653	1.837 ± 2.286	12.889 ± 14.05	3.391 ± 3.563	25	30.769	40	30	5.882
*SERPINF1*	17	7.882 ± 5.372	−3.386 ± 2.513	2.186 ± 2.874	32.794 ± 38.085	7.038 ± 6.988	18.75	27.273	50	75	0
*FKBP10*	10	12.95 ± 9.051	−5.965 ± 4.414	6.183 ± 5.636	18.722 ± 31.214	1.792 ± 1.355	40	25	11.111	71.429	0
*p* value			0.147	0.08	0.078	**<0.001**	0.759	0.19	**<0.001**	**<0.001**	0.086
AD OI	241	16.873 ± 11.917	−4.476 ± 4.156	6.171 ± 7.971	17.129 ± 22.201	2.208 ± 2.609	23.707	50.909	91.556	68.75	18.905
AR OI	57	9.118 ± 8.272	−4.359 ± 3.798	2.705 ± 3.741	21.056 ± 29.47	4.238 ± 4.939	28.571	35	43.636	54.902	2.439
*p* value		**<0.001**	0.858	**<0.001**	0.361	**0.004**	0.448	0.071	**<0.001**	0.059	**0.009**
collagen I quantitative variant	32	16.094 ± 12.721	−1.128 ± 2.011	5.978 ± 6.402	11.823 ± 12.518	1.794 ± 1.468	16.129	78.947	100	50	22.222
collagen I structural variant	162	17.95 ± 11.83	−5.52 ± 4.324	6.449 ± 8.173	19.44 ± 25.315	2.233 ± 2.87	26.452	43.243	90.132	76	18.657
*p* value		0.45	**<0.001**	0.724	**0.014**	0.218	0.224	**0.004**	0.068	**0.004**	0.668
collagen I Gly substitution	160	18.39 ± 12.393	−5.376 ± 4.361	6.787 ± 8.441	18.455 ± 24.183	2.113 ± 2.581	27.097	46.364	91.447	73.826	17.91
collagen I other substitution	66	14.167 ± 9.945	−2.486 ± 2.751	4.67 ± 5.675	15.105 ± 18.549	2.415 ± 2.788	12.903	60.465	96.552	63.333	22.642
*p* value		**0.008**	**<0.001**	**0.034**	0.28	0.467	**0.025**	0.117	0.199	0.131	0.46
α1(I) chain structural variant	64	19.095 ± 12.124	−5.928 ± 4.975	6.631 ± 8.139	19.754 ± 24.708	2.605 ± 4.131	29.31	40.476	93.103	77.193	26.923
α2(I) chain structural variant	98	17.214 ± 11.641	−5.262 ± 3.863	6.337 ± 8.235	19.245 ± 25.813	2.002 ± 1.654	24.742	44.928	88.298	75.269	13.415
*p* value		0.331	0.392	0.828	0.903	0.289	0.533	0.646	0.335	0.789	0.05
α1(I) chain Gly substitution	68	19.493 ± 12.961	−5.471 ± 4.971	6.969 ± 8.12	18.648 ± 23.72	2.384 ± 3.55	29.688	46.667	95.312	70.968	23.214
α2(I) chain Gly substitution	92	17.587 ± 11.971	−5.306 ± 3.88	6.658 ± 8.701	18.317 ± 24.639	1.923 ± 1.583	25.275	46.154	88.636	75.862	14.103
*p* value		0.346	0.828	0.82	0.933	0.337	0.543	0.958	0.146	0.503	0.175

Significant differences (*p* < 0.05) in bold font; DI, dentinogenesis imperfecta; SD, standard deviation.

## Data Availability

Clinical manifestations and causative variants from the 298 families have been submitted to the Chinese Osteogenesis Imperfecta Mutation Database (http://coimd.ncmi.cn; accessed on 8 September 2022).

## Supplementary Materials

The following supporting information can be downloaded at: https://www.mdpi.com/article/10.3390/genes16040416/s1, Table S1: Clinical and genetic characteristics of OI patients. Table S2: List of primers used for qPCR and Gap-PCR. Table S3: Characteristics of OI patients in different age groups.
